# Were VCF patients at higher risk of mortality following the 2009 publication of the vertebroplasty “sham” trials?

**DOI:** 10.1007/s00198-017-4281-z

**Published:** 2017-10-24

**Authors:** K. L. Ong, D. P. Beall, M. Frohbergh, E. Lau, J. A. Hirsch

**Affiliations:** 1 Exponent, Inc., 3440 Market St, Suite 600, Philadelphia, PA USA; 2 Oklahoma Spine, Edmond, OK USA; 3 Exponent, Inc., Menlo Park, CA USA; 4 Massachusetts General Hospital, Boston, MA USA

**Keywords:** Balloon kyphoplasty, Mortality, Vertebral augmentation, Vertebral compression fracture, Vertebroplasty

## Abstract

**Summary:**

The 5-year period following 2009 saw a steep reduction in vertebral augmentation volume and was associated with elevated mortality risk in vertebral compression fracture (VCF) patients. The risk of mortality following a VCF diagnosis was 85.1% at 10 years and was found to be lower for balloon kyphoplasty (BKP) and vertebroplasty (VP) patients.

**Introduction:**

BKP and VP are associated with lower mortality risks than non-surgical management (NSM) of VCF. VP versus sham trials published in 2009 sparked controversy over its effectiveness, leading to diminished referral volumes. We hypothesized that lower BKP/VP utilization would lead to a greater mortality risk for VCF patients.

**Methods:**

BKP/VP utilization was evaluated for VCF patients in the 100% US Medicare data set (2005–2014). Survival and morbidity were analyzed by the Kaplan-Meier method and compared between NSM, BKP, and VP using Cox regression with adjustment by propensity score and various factors.

**Results:**

The cohort included 261,756 BKP (12.6%) and 117,232 VP (5.6%) patients, comprising 20% of the VCF patient population in 2005, peaking at 24% in 2007–2008, and declining to 14% in 2014. The propensity-adjusted mortality risk for VCF patients was 4% (95% CI, 3–4%; *p* < 0.001) greater in 2010–2014 versus 2005–2009. The 10-year risk of mortality for the overall cohort was 85.1%. BKP and VP cohorts had a 19% (95% CI, 19–19%; *p* < 0.001) and 7% (95% CI, 7–8%; *p* < 0.001) lower propensity-adjusted 10-year mortality risk than the NSM cohort, respectively. The BKP cohort had a 13% (95% CI, 12–13%; *p* < 0.001) lower propensity-adjusted 10-year mortality risk than the VP cohort.

**Conclusions:**

Changes in treatment patterns following the 2009 VP publications led to fewer augmentation procedures. In turn, the 5-year period following 2009 was associated with elevated mortality risk in VCF patients. This provides insight into the implications of treatment pattern changes and associated mortality risks.

## Introduction

Osteoporosis affects up to 12 million older adults in the USA, with an additional 47 million affected by low bone mass [1]. The number of older adults with osteoporosis or low bone mass is expected to increase in the USA by about 17 million (32%) from 2010 to 2030 [2]. Spine fracture prevalence is approximately 5.4% in adults aged 40 years and older, increasing to 18% in those 80 years and older [3]. Vertebral compression fractures (VCFs) can lead to a downward spiral of symptoms and morbidity, from pain and disability to impaired pulmonary and respiratory function [4]. There are also associated mortality risks, with up to 72% mortality rate at 5 years [5] and 90% at 7 years [6].

Narcotic analgesics, back braces, and immobilization are common non-surgical means for VCF treatment, but may be poorly tolerated in elderly patients with side effects, such as constipation and increased risk of falls [7, 8]. Patients can also become dependent on opioids, which can be dangerous if misused [9]. Alternatively, surgical interventions with vertebroplasty (VP) or balloon kyphoplasty (BKP) can provide improved pain relief, functional recovery, and health-related quality of life [8, 10–13]. Moreover, lower mortality risks have been reported for augmentation over non-surgically managed (NSM) patients in the majority of claims-based studies [14–18]. A study of over one million elderly Medicare patients with VCFs, including over 75,000 VP and 140,000 BKP patients, described a 25 and 55% elevated mortality risk for NSM than VP and BKP, respectively [16].

In 2009, controversy sparked over the effectiveness of VP with publications by Kallmes et al. and Buchbinder et al., with approximately 200 patients in aggregate [19, 20]. Both identified no benefit in pain or functional improvements for VP over a “sham” procedure that included the periosteal injection of local anesthetic [19, 20]. Yet periosteal local anesthetic infiltration can have a treatment effect, making it an active control [21]; this is one of the important differences in trial design between the 2009 trials and a more recent VP trial [8, 22]. A physician survey later showed that at least one of these two “sham” control studies was directly linked to reduced enthusiasm for VP referrals, even though most still felt that VP was an effective procedure in appropriate patients [23]. Nonetheless, the period following those publications exhibited diminished volumes of vertebral augmentation [23–26]. When taken in context of the trends in utilization of percutaneous interventional procedures for managing spinal pain [27, 28], the decrease is almost certainly the direct or indirect result of the two “sham” control studies.

With questions raised about the effectiveness of VP and the corresponding reductions in number of patients treated, this study addressed the following research questions: (1) What is the utilization of BKP/VP in the US elderly patient population? (2) Did the mortality risk for VCF patients differ between 2010 and 2014 and 2005–2009? (3) Are there differences in mortality and morbidity risks between BKP/VP and NSM patients?

## Methods

The 100% inpatient/outpatient Medicare claims data (2005–2014) was used to identify newly diagnosed VCF patients (International Classification of Diseases, Ninth Revision, Clinical Modification (ICD-9-CM) codes 733.13, 805.0, 805.2, 805.4, 805.6, and 805.8). The first VCF diagnosed in the study period was used; patients were required to have at least 12-month claims history prior to the VCF diagnosis to confirm a VCF-free period. Patients with BKP/VP in the 12 months before the index VCF were excluded. Those younger than 65 years old were also excluded due to potential confounding factors from their Medicare eligibility (certain disabilities, permanent kidney failure, amyotrophic lateral sclerosis, etc.). Patients enrolled in a Health Maintenance Organization plan (such as, Medicare Advantage plan), not enrolled in both Parts A and B of Medicare, not residing in the 50 states, and without 12 months of claims history prior to the VCF diagnosis were also excluded due to potential incompleteness in their claims history. The annual VCF incidence was determined from the number of newly diagnosed VCFs and Medicare enrollees. The patients were stratified into NSM, BKP, and VP cohorts. BKP/VP cohorts were those who underwent augmentation within the first year of the VCF diagnosis; those who underwent fusion surgery between the VCF diagnosis and BKP/VP were excluded. The NSM cohort comprised of patients who did not undergo augmentation or fusion during the study period, and those who only underwent augmentation or fusion 1+ years after the index VCF diagnosis. BKP was identified using ICD-9-CM code 81.66 or Current Procedural Terminology (CPT) codes 22289 and 22523–22525, while VP was identified using ICD-9-CM 81.65 or CPT codes 22520–22522. Spine fusion was identified using ICD-9-CM codes 81.61, 84.51, 81.00–81.08, 81.30–81.39 or CPT codes 22532–22534, 22548, 22554, 22556, 22558, 22585, 22590, 22595, 22600, 22610, 22612, 22614, 22630, 22632, 22800, 22802, 22804, 22808, 22810, 22812, 22840–22847, 22849, and 22851. This study was based on publicly available data sets, did not use private health identifiable information, and did not represent human subject research, and therefore did not require oversight by our institutional review boards.

Mortality was the primary outcome, based on the date of death from the Medicare denominator file, which contains enrollment and eligibility information. Mortality with pneumonia diagnosed as a principal diagnosis or any (principal or secondary) diagnosis within 90 days prior to death was also determined. Morbidity conditions examined in this study included myocardial infarction/cardiac complications (ICD-9-CM codes 410, 997.1), deep venous thrombosis (451, 453), infection (diagnosis 996.67, 999.3, 998.5; procedure, ICD-9-CM codes 77.69, 86.22, 86.28 or CPT codes 10180, 22010, 22015), pulmonary embolism (415.11, 415.19), pneumonia (480–487), urinary tract infection (595.0, 595.2, 595.3, 595.8, 595.89, 595.9, 599.0, 996.64), pulmonary/respiratory complications (490–496, 510–519), and readmissions. All outcomes were evaluated at up to 10 years follow-up, while readmissions were evaluated up to 1 year to limit the effects from other unrelated interventions. The length of stay (LOS) and discharge status following the first VCF hospitalization for the NSM cohort and the index augmentation surgery for the BKP and VP cohorts (inpatients) were compared.

Mortality for VCF patients between the 2005–2009 and 2010–2014 time periods was compared using multivariate Cox regression, adjusting for propensity score, gender, age, race, census region, socioeconomic status, comorbidities, type of fracture (traumatic or pathologic), fracture location (cervical, thoracic, lumbar, sacrum), initial VCF diagnosis site of service (inpatient, outpatient), physician specialty for initial VCF diagnosis, and treatment group (NSM, BKP, VP). Socioeconomic status was based on whether the patient’s Medicare premiums/deductibles were state subsidized (Medicare buy-in status), as well as the per capita income for the patient’s county of residence. Comorbidities were determined using the Charlson score [29] and the diagnosis of 12 specific comorbid conditions in the 12 months prior to the VCF. The specific comorbid conditions comprised: (1) arterial disease (ICD-9-CM codes 440–448), (2) chronic obstructive pulmonary disease (490–496), 3) cancer (140–176, 179–208, 210–239, V10), (4) diabetes (250), (5) hip fracture (820), (6) hypertensive disease (401–405), (7) ischemic heart disease (410–414), (8) other heart disease (420–429), (9) pneumonia (480–487, V12.6), (10) pulmonary heart disease (415–417, V12.5), (11) stroke (430–438), and (12) wrist fracture (813.4, 813.5, 814.0, 814.1). Propensity score was derived for the probability of undergoing augmentation, using logistic regression conditional on gender, age, race, census region, socioeconomic status, comorbidities, type of fracture, diagnosis of osteoporosis, fracture location, initial VCF diagnosis site of service, physician specialty for initial VCF diagnosis, year, and two-way interactions for all the above covariates (except per capita income and physician specialty). Statistical comparisons of the mortality and morbidity conditions were also compared between the study cohorts, using propensity-adjusted, multivariate Cox regression. The covariates were the same as above, except time period (2005–2009 vs. 2010–2015) was replaced with year as a continuous variable.

## Results

**Fig. 1:**
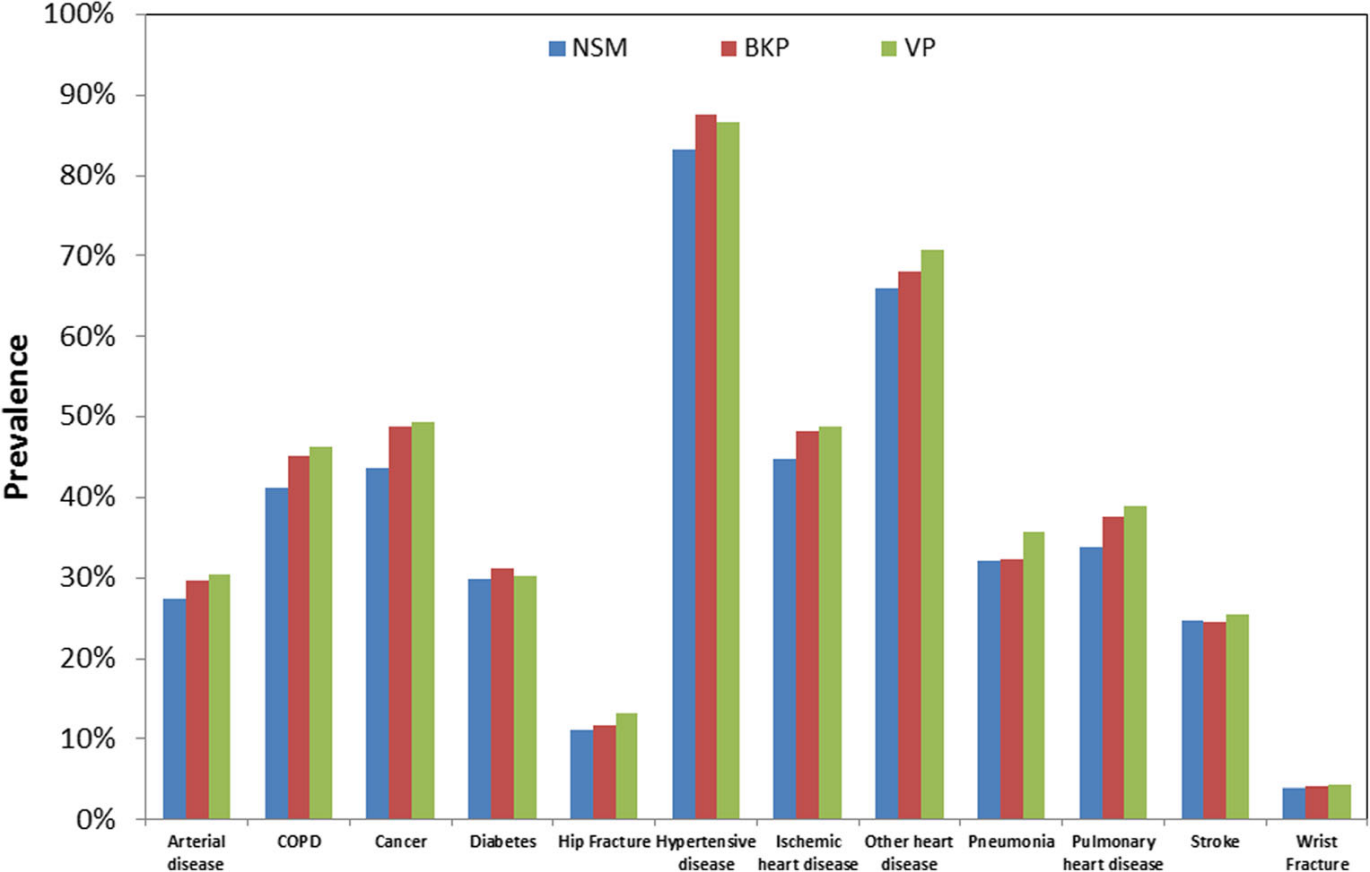
Prevalence of comorbidities in the 12 months prior to VCF diagnosis

Our study identified 2,129,769 newly diagnosed VCF patients. VCF prevalence was 239,325 in 2005, and then declined to 200,595 in 2010, before increasing to 209,337 in 2014. After accounting for the annual Medicare population, VCF incidence decreased from 74.7 to 62.0 VCFs per ten thousand enrollees between 2005 and 2014. During 2005 to 2008, VP volume ranged between 16,258 and 16,858 annually, but declined from 15,742 procedures in 2009 to 8419 procedures in 2014. BKP volume increased from 16,704 in 2005 to 33,648 in 2008 before experiencing a less steep volume reduction from 32,715 in 2009 to 29,679 in 2014. Vertebral augmentation patients comprised 20% of the VCF population in 2005, peaked at 24% in 2007–2008, and declined to 14% in 2014. After excluding patients who were treated with fusion within a year after VCF, including between VCF and BKP/VP, the final study cohort included 2,077,944 VCF patients (*n* = 261,756 BKP (12.6%) and 117,232 VP (5.6%)). Among the VCF study cohort, hypertensive disease and other heart disease were the most commonly diagnosed comorbidities (Fig. 1). Close to half the patients were also diagnosed with ischemic heart disease and cancer. NSM patients did not have a higher prevalence of comorbidities than the augmented patients. Baseline demographics of the three cohorts are provided in Table 1.

**Table 1:** Baseline demographics of NSM, BKP, and VP patients

	Non-operated (*n* = 1,698,956) (%)	Balloon kyphoplasty (*n* = 261,756) (%)	Vertebroplasty (*n* = 117,232) (%)
% female	70.2	72.5	73.1
White	91.8	94.6	95.1
Black	3.2	1.6	1.5
Others	4.9	3.8	3.4
65–69 years old	12.3	11.8	10.5
70–74 years old	14.7	16.2	14.8
75–79 years old	18.8	21.8	21.0
80–84 years old	22.4	24.7	24.9
≥ 85 years old	31.9	25.5	28.9
Midwest	26.4	23.3	34.9
Northeast	18.8	13.7	9.8
South	36.9	50.2	38.8
West	17.9	12.8	16.5
Charlson index 0	32.7	32.8	32.8
1–2	38.5	39.0	38.8
3–4	16.6	16.5	16.7
≥ 5	12.2	11.7	11.7
% with Medicare buy-in	17.4	12.3	11.7

**Fig. 2:**
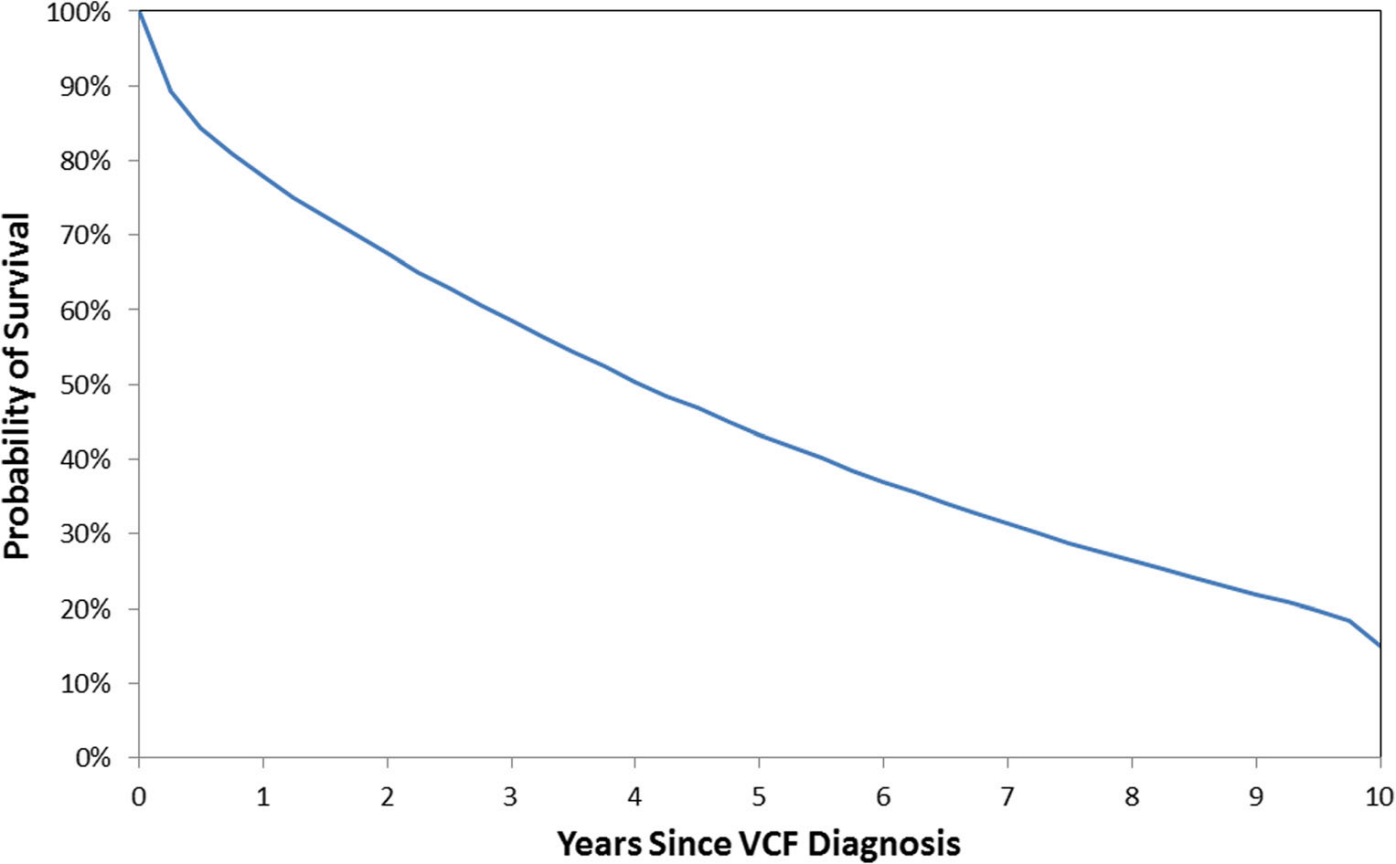
Overall survival of VCF patients

**Fig. 3:**
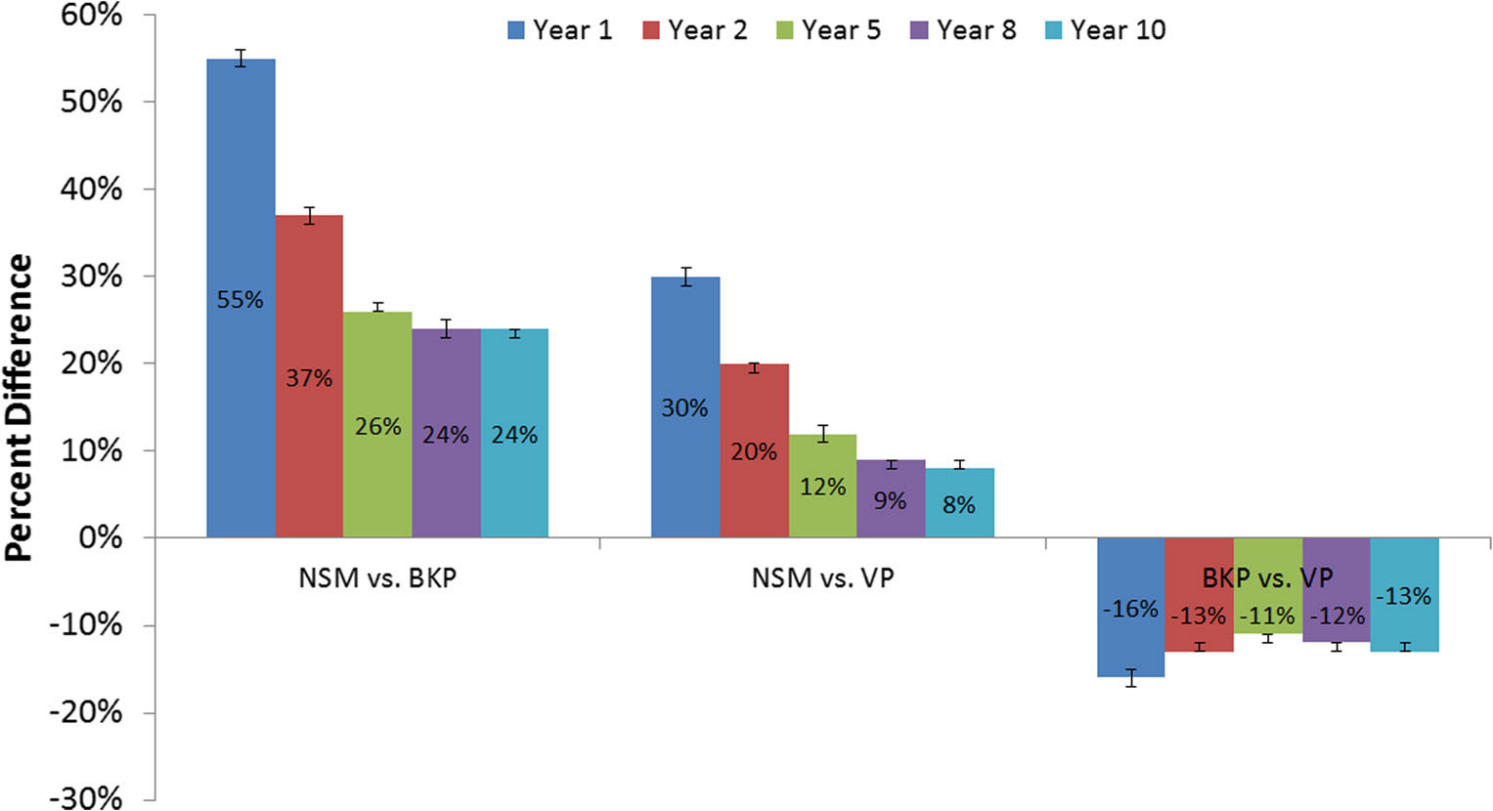
Relative risk of mortality (propensity-adjusted) between NSM, BKP, and VP cohorts (*p* < 0.001 for all)

Mortality risk for the overall VCF cohort was 85.1% (95% CI, 84.7–85.5%) at 10 years (Fig. 2). When comparing time periods, the propensity-adjusted mortality risk for VCF patients was 4% (95% CI, 3–4%; *p* < 0.001) greater in 2010–2014 than 2005–2009. Additional factors associated with increased mortality risk included older age, higher Charlson score, cervical or thoracic fractures, lower socioeconomic status (household income and buy-in status), those diagnosed in an inpatient setting, Caucasians, patients in the South, males, non-surgical managed patients, diagnosed pathologic fractures, as well as history of other heart diseases, and pneumonia (all *p* < 0.001). Following stratification by treatment group, the NSM cohort had 24% (95% CI, 23–24%; *p* < 0.001) and 8% (95% CI, 8–9%; *p* < 0.001) higher propensity-adjusted 10-year mortality risks than the BKP and VP cohorts, respectively (Fig. 3). In other words, the BKP and VP cohorts had a 19% (95% CI, 19–19%; *p* < 0.001) and 7% (95% CI, 7–8%; *p* < 0.001) lower propensity-adjusted 10-year mortality risk than the NSM cohort, respectively. The BKP cohort had a 13% (95% CI, 12–13%; *p* < 0.001) lower propensity-adjusted 10-year mortality risk than the VP cohort. These were still statistically different at earlier time points. For 10-year mortality risk with pneumonia as a principal diagnosis within 90 days prior to death, the NSM cohort had 19% (95% CI, 17–20%; *p* < 0.001) and 8% (95% CI, 6–10%; *p* < 0.001) higher risks than the BKP and VP cohorts, respectively, while the BKP cohort had a 9% (95% CI, 7–10%) lower risk than the VP cohort. For 10-year mortality risk with pneumonia as a principal/secondary diagnosis within 90 days prior to death, the NSM cohort had 21% (95% CI, 20–22%; *p* < 0.001) and 3% (95% CI, 2–4%; *p* < 0.001) higher risks than the BKP and VP cohorts, respectively, while the BKP cohort had a 15% (95% CI, 14–16%; *p* < 0.001) lower risk than the VP cohort.

**Fig. 4:**
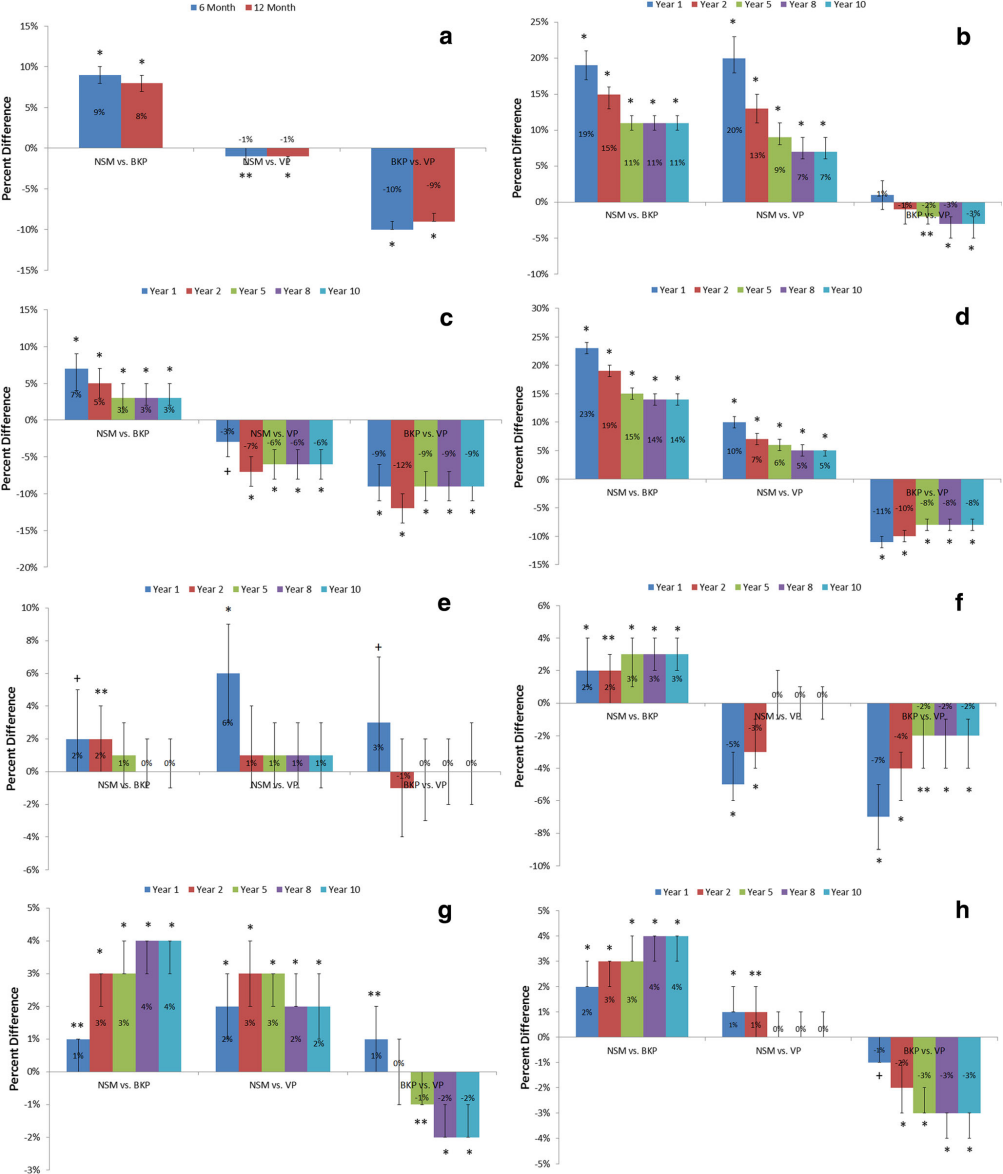
Relative propensity-adjusted risks of readmission (**a**), cardiac complications (**b**), pulmonary embolism (**c**), pneumonia (**d**), infection (**e**), DVT (**f**), UTI (**g**), and pulmonary/respiratory complications (**h**) between NSM, BKP, and VP cohorts (*p < 0.001; **p < 0.01; +*p* < 0.05)

The propensity-adjusted risk of readmissions and morbidities including cardiac complications, pulmonary embolism, pneumonia, deep venous thrombosis, urinary tract infection, and pulmonary/respiratory complications were significantly higher for the NSM cohort than BKP cohort at all time points (Fig. 4). At 1 year, outcomes with at least 10% greater risk for NSM than BKP patients were cardiac complications (19%; 95% CI, 17–21%; *p* < 0.001) and pneumonia (23%; 95% CI, 22–24%; *p* < 0.001). Compared to the VP cohort, the NSM cohort also had significantly higher propensity-adjusted risk of cardiac complications, pneumonia, and urinary tract infection at all time points, but had significantly lower risk of pulmonary embolism and readmission. The propensity-adjusted risk of readmission, pulmonary embolism, pneumonia, deep venous thrombosis, and pulmonary/respiratory complications were significantly lower for BKP than VP cohorts at all time points. The mean LOS for hospitalized NSM, BKP, and VP patients were 5.2 days (± 4.5 days), 5.4 days (± 5.1 days), and 6.6 days (± 5.5 days), respectively. Although the average LOS appeared similar between the NSM and BKP cohorts, after adjusting for various patient and clinical factors, BKP patients were found to have significantly longer LOS by 18% (95% CI, 18–19%; *p* < 0.001) (Table 2). The VP cohort also had significantly longer adjusted LOS than the NSM cohort by 36% (95% CI, 35–37%; *p* < 0.001), while the BKP cohort had shorter adjusted LOS than the VP cohort by 13% (95% CI, 13–13%; *p* < 0.001). More than half of the BKP patients (56.9%) were discharged to home, when compared to the VP (47.0%) and NSM (33.7%) cohorts. Nearly half of the NSM patients were discharged to skilled nursing facilities (48.0%), compared to 31.0% of BKP and 39.6% of VP patients. After adjusting for patient and clinical factors, BKP patients were more than twice as likely to be discharged to home than NSM patients (odds ratio 2.27; 95% CI, 2.20–2.35; *p* < 0.001) (Table 2). BKP patients were 22% (95% CI, 19–25%; *p* < 0.001) more likely to be discharged to home than VP patients, while VP patients were, in turn, 86% (95% CI, 80–93%; *p* < 0.001) more likely to be discharged to home than NSM patients.

**Table 2:** Comparison of LOS and discharge to home (propensity-adjusted)

Treatment group	Reference group	LOS ratio	Lower limit	Upper limit	*p* value
BKP	Non-operated	1.18	1.18	1.19	< 0.001
VP	Non-operated	1.36	1.35	1.37	< 0.001
BKP	VP	0.87	0.87	0.87	< 0.001
Treatment group	Reference group	Discharge-to-home ratio	Lower limit	Upper limit	*p* value
BKP	Non-operated	2.27	2.20	2.35	< 0.001
VP	Non-operated	1.86	1.80	1.93	< 0.001
BKP	VP	1.22	1.19	1.25	< 0.001

## Discussion

This analysis of over two million VCF patients in the US Medicare population showed that the 10-year mortality risk was exceptionally high at 85%. The NSM cohort had 24 and 8% greater 10-year mortality risks than the BKP and VP cohorts, respectively, and the BKP cohort also had a 13% lower 10-year mortality risk than the VP cohort. Vertebral augmentation utilization peaked at 24% in 2007–2008 and then declined to 14% in 2014. The mortality risk for VCF patients was also significantly greater in 2010–2014 than 2005–2009.

Vertebral augmentation in the Medicare population was noted to decline from 2009 onwards, although this impacted VP more dramatically. Researchers [23, 25] have attributed diminished VP volumes to the controversy sparked by the 2009 “sham” control publications [19, 20]. Others [30] have also pointed to insurance coverage changes or recommendations from professional societies, such as the American Academy of Orthopaedic Surgeons (AAOS) guidelines, which were impacted by the two aforementioned studies [31]. Despite the reduced volume, augmentation continues to be offered to many patients, which could reflect the difficulties faced by clinicians in reconciling the findings from the 2009 “sham” control papers and their clinical experience of patient improvements [32]. There is substantial evidence supporting the use of augmentation [8, 10–13, 33]. A lead site in one of the 2009 “sham” control studies also continued to perform VP relatively frequently, indicating “our [their] own belief in the efficacy of the procedure outweighs its risks” [25].

Consistent with earlier time periods [24, 26, 30], this present analysis of the Medicare population showed continued decline in BKP/VP utilization from 2009 through 2014, which indicated that relatively more VCF patients in the latter half of the study period were being non-surgically managed. Since previous studies have predominantly shown survival benefits from augmentation [14–18], patients in the latter half of the study period may be at higher risk of death. This was confirmed with the significantly higher 5-year mortality risk for VCF patients from 2010 to 2014 than 2005–2009. Moreover, VP and BKP cohorts had significantly lower propensity-adjusted mortality risks than the NSM cohort at up to 10 years, a much longer follow-up than previous studies [14–18]. Those previous mortality studies also showed improved survival for BKP/VP over NSM [14–17], except for one by McCullough et al. [18], who reported lower adjusted mortality risks at 30 days and 1 year for augmented patients, but only at 30 days after propensity score matching.

This study found that LOS was longer for hospitalized augmented patients than NSM patients, but the augmented patients had a higher likelihood of being discharged to home. VP patients were also more likely to have longer LOS and less likely to be discharged to home than BKP. Notably, the opposite trends in the LOS and home discharge rates appears to reflect a shifting of the NSM patients from the inpatient to other facilities, and do not reflect recovery of the patients. Any perceived cost savings from the 0.2 days shorter LOS, on average, for the NSM cohort over the BKP cohort was outweighed by close to twice as few NSM patients being discharged to home. In contrast to this study, Chen and coworkers [14] observed shorter LOS for BKP patients than NSM patients, but this could be due to the different study periods. The Chen study utilized Medicare data from 2006, whereas this analysis observed some temporal changes in LOS during the study period; the average LOS for the BKP cohort increased between 2005 and 2009 and then remained relatively stable. Both the Chen study and this present analysis reported LOS for inpatients, which likely reflects the experience for sicker patients, but did not include outpatients, which would have lowered the overall LOS.

This study has several limitations. Although the present analysis focused on mortality and morbidity risks, outcomes, such as pain relief or quality of life, could not be assessed due to inherent limitations of claims data. The effects of several comorbidities, including previous diagnosis of hip or wrist fractures, were considered in the analysis, but other clinical variables or baseline health conditions, such as fracture severity and severity of the underlying osteoporosis, which are not captured in the database, may have potential confounding effects. This study was also unable to determine the criteria for referrals to BKP and VP compared to NSM. Because the NSM cohort was discharged to nursing facilities at a significantly higher rate than BKP and VP cohorts, the present analysis could not accurately assess the LOS between the three cohorts. The cause of death is unknown in the data set, but various morbidities and mortality with pneumonia diagnosed in the 90 days prior to death were used to provide some insight into the health status leading to expiration. There may be potential for selection bias due to the observational study design, which this study attempted to minimize by controlling for a large number of confounding factors and propensity scoring. On the other hand, this study provides real-world outcomes for a large population of over two million VCF patients, which is not feasible through a randomized controlled trial. The Medicare claims data also provides consistency of follow-up because loss of “enrollment” would only occur through death.

## Summary

There has been extensive debate following publication of two 2009 “sham” control studies. Many medical societies have supported the continued use of augmentation as a safe and efficacious procedure for symptomatic VCFs [34] or as being reasonable options for selected patients [35], but the AAOS strongly recommended against VP and provided limited recommendation for BKP [31]. National treatment guidelines or technology assessments have also been mixed [36–38]. Based on this present analysis of over two million VCF patients in the Medicare population, publication of the 2009 “sham” control studies likely resulted in lower augmentation utilization, and in turn, the 5-year period following 2009 was associated with elevated mortality risk in VCF patients. These findings provide real-world insight into the implications of shifts in treatment patterns and associated mortality risks for VCF patients.

## References

[1] Looker AC, Sarafrazi Isfahani N, Fan B, Shepherd JA (2017) Trends in osteoporosis and low bone mass in older US adults, 2005-2006 through 2013-2014. Osteoporos Int. 10.1007/s00198-017-3996-1 10.1007/s00198-017-3996-1 PMC7891684 28315954

[2] Wright NC, Looker AC, Saag KG, Curtis JR, Delzell ES, Randall S, Dawson-Hughes B (2014). The recent prevalence of osteoporosis and low bone mass in the United States based on bone mineral density at the femoral neck or lumbar spine. J Bone Mineral Res.

[3] Cosman F, Krege JH, Looker AC, Schousboe JT, Fan B, Sarafrazi Isfahani N, Shepherd JA, Krohn KD, Steiger P, Wilson KE, Genant HK (2017) Spine fracture prevalence in a nationally representative sample of US women and men aged ≥40 years: results from the National Health and Nutrition Examination Survey (NHANES) 2013–2014. Osteoporos Int. 10.1007/s00198-017-3948-9 10.1007/s00198-017-3948-9 PMC7422504 28175980

[4] Old JL, Calvert M (2004). Vertebral compression fractures in the elderly. Am Fam Physician.

[5] Johnell O, Kanis JA, Oden A, Sernbo I, Redlund-Johnell I, Petterson C, De Laet C, Jonsson B (2004). Mortality after osteoporotic fractures. Osteoporos Int.

[6] Lau E, Ong K, Kurtz S, Schmier J, Edidin A (2008). Mortality following the diagnosis of a vertebral compression fracture in the Medicare population. J Bone Joint Surg Am.

[7] Goldstein CL, Chutkan NB, Choma TJ, Orr RD (2015). Management of the elderly with vertebral compression fractures. Neurosurgery.

[8] Clark W, Bird P, Gonski P, Diamond TH, Smerdely P, McNeil HP, Schlaphoff G, Bryant C, Barnes E, Gebski V (2016). Safety and efficacy of vertebroplasty for acute painful osteoporotic fractures (VAPOUR): a multicentre, randomised, double-blind, placebo-controlled trial. Lancet.

[9] Wilson-Poe AR, Moron JA (2017) The dynamic interaction between pain and opioid misuse. Br J Pharmacol. 10.1111/bph.13873 10.1111/bph.13873 PMC6016619 28602044

[10] Berenson J, Pflugmacher R, Jarzem P, Zonder J, Schechtman K, Tillman JB, Bastian L, Ashraf T, Vrionis F, Cancer Patient Fracture Evaluation I (2011). Balloon kyphoplasty versus non-surgical fracture management for treatment of painful vertebral body compression fractures in patients with cancer: a multicentre, randomised controlled trial. The Lancet Oncology.

[11] Boonen S, Van Meirhaeghe J, Bastian L, Cummings SR, Ranstam J, Tillman JB, Eastell R, Talmadge K, Wardlaw D (2011). Balloon kyphoplasty for the treatment of acute vertebral compression fractures: 2-year results from a randomized trial. J Bone Mineral Res.

[12] Klazen CA, Lohle PN, de Vries J, Jansen FH, Tielbeek AV, Blonk MC, Venmans A, van Rooij WJ, Schoemaker MC, Juttmann JR, Lo TH, Verhaar HJ, van der Graaf Y, van Everdingen KJ, Muller AF, Elgersma OE, Halkema DR, Fransen H, Janssens X, Buskens E, Mali WP (2010). Vertebroplasty versus conservative treatment in acute osteoporotic vertebral compression fractures (Vertos II): an open-label randomised trial. Lancet.

[13] Wardlaw D, Cummings SR, Van Meirhaeghe J, Bastian L, Tillman JB, Ranstam J, Eastell R, Shabe P, Talmadge K, Boonen S (2009). Efficacy and safety of balloon kyphoplasty compared with non-surgical care for vertebral compression fracture (FREE): a randomised controlled trial. Lancet.

[14] Chen AT, Cohen DB, Skolasky RL (2013). Impact of nonoperative treatment, vertebroplasty, and kyphoplasty on survival and morbidity after vertebral compression fracture in the medicare population. J Bone Joint Surg Am.

[15] Edidin AA, Ong KL, Lau E, Kurtz SM (2011). Mortality risk for operated and nonoperated vertebral fracture patients in the medicare population. J Bone Mineral Res.

[16] Edidin AA, Ong KL, Lau E, Kurtz SM (2015). Morbidity and mortality after vertebral fractures: comparison of vertebral augmentation and nonoperative management in the Medicare population. Spine.

[17] Lange A, Kasperk C, Alvares L, Sauermann S, Braun S (2014). Survival and cost comparison of kyphoplasty and percutaneous vertebroplasty using German claims data. Spine.

[18] McCullough BJ, Comstock BA, Deyo RA, Kreuter W, Jarvik JG (2013). Major medical outcomes with spinal augmentation vs conservative therapy. JAMA Intern Med.

[19] Buchbinder R, Osborne RH, Ebeling PR, Wark JD, Mitchell P, Wriedt C, Graves S, Staples MP, Murphy B (2009). A randomized trial of vertebroplasty for painful osteoporotic vertebral fractures. N Engl J Med.

[20] Kallmes DF, Comstock BA, Heagerty PJ, Turner JA, Wilson DJ, Diamond TH, Edwards R, Gray LA, Stout L, Owen S, Hollingworth W, Ghdoke B, Annesley-Williams DJ, Ralston SH, Jarvik JG (2009). A randomized trial of vertebroplasty for osteoporotic spinal fractures. N Engl J Med.

[21] Wilson DJ, Owen S, Corkill RA (2011). Facet joint injections as a means of reducing the need for vertebroplasty in insufficiency fractures of the spine. Eur Radiol.

[22] Hirsch JA, Chandra RV (2016). Resurrection of evidence for vertebroplasty?. Lancet.

[23] Lindsey SS, Kallmes DF, Opatowsky MJ, Broyles EA, Layton KF (2013). Impact of sham-controlled vertebroplasty trials on referral patterns at two academic medical centers. PRO.

[24] Hirsch JA, Chandra RV, Pampati V, Barr JD, Brook AL, Manchikanti L (2016) Analysis of vertebral augmentation practice patterns: a 2016 update. J Neurointerventional Surg. 10.1136/neurintsurg-2016-012767 10.1136/neurintsurg-2016-012767 27799375

[25] Luetmer MT, Kallmes DF (2011). Have referral patterns for vertebroplasty changed since publication of the placebo-controlled trials?. AJNR Am J Neuroradiol.

[26] Manchikanti L, Pampati V, Hirsch JA (2013). Analysis of utilization patterns of vertebroplasty and kyphoplasty in the Medicare population. J Neurointerventional Surg.

[27] Manchikanti L, Pampati V, Hirsch JA (2016). Utilization of interventional techniques in managing chronic pain in Medicare population from 2000 to 2014: an analysis of patterns of utilization. Pain Physician.

[28] Manchikanti L, Pampati V, Hirsch JA (2016). Retrospective cohort study of usage patterns of epidural injections for spinal pain in the US fee-for-service Medicare population from 2000 to 2014. BMJ Open.

[29] Charlson ME, Pompei P, Ales KL, MacKenzie CR (1987). A new method of classifying prognostic comorbidity in longitudinal studies: development and validation. J Chronic Dis.

[30] Goz V, Errico TJ, Weinreb JH, Koehler SM, Hecht AC, Lafage V, Qureshi SA (2015). Vertebroplasty and kyphoplasty: national outcomes and trends in utilization from 2005 through 2010. Spine J: Off J North Am Spine Soc.

[31] American Academy of Orhopaedic Surgeons (2010) The treatment of symptomatic osteoporotic spinal compression fractures: guideline and evidence report. http://www.aaos.org/research/guidelines/SCFguideline.pdf. Accessed June 1, 2017

[32] McDonald RJ, Lane JI, Diehn FE, Wald JT (2017). Percutaneous vertebroplasty: overview, clinical applications, and current state. Appl Radiol.

[33] Beall DP, F. CM, Thomas SM, Easton R, Talati S, Goodman B, Datta D, Webb JR, Linville D EVOLVE (2017) A prospective multicenter evaluation of quality of life, pain & activities of daily living outcomes for balloon kyphoplasty in the treatment of medicare patients with vertebral compression fractures. In: Evidence-based spine interventions seminar, Palm Springs

[34] Barr JD, Jensen ME, Hirsch JA, JK MG, Barr RM, Brook AL, Meyers PM, Munk PL, Murphy KJ, O'Toole JE, Rasmussen PA, Ryken TC, Sanelli PC, Schwartzberg MS, Seidenwurm D, Tutton SM, Zoarski GH, Kuo MD, Rose SC, Cardella JF, Society of Interventional R, American Association of Neurological S, Congress of Neurological S, American College of R, American Society of N, American Society of Spine R, Canadian Interventional Radiology A, Society of Neurointerventional S (2014). Position statement on percutaneous vertebral augmentation: a consensus statement developed by the Society of Interventional Radiology (SIR), American Association of Neurological Surgeons (AANS) and the Congress of Neurological Surgeons (CNS), American College of Radiology (ACR), American Society of Neuroradiology (ASNR), American Society of Spine Radiology (ASSR), Canadian Interventional Radiology Association (CIRA), and the Society of NeuroInterventional Surgery (SNIS). J Vasc Int Radiol: JVIR.

[35] Chandra RV, Meyers PM, Hirsch JA, Abruzzo T, Eskey CJ, Hussain MS, Lee SK, Narayanan S, Bulsara KR, Gandhi CD, Do HM PCJ, Albuquerque FC, Frei D, Kelly ME, Mack WJ, Pride GL, Jayaraman MV, Society of NeuroInterventional S (2014). Vertebral augmentation: report of the Standards and Guidelines Committee of the Society of NeuroInterventional Surgery. J Neurointerventional Surg.

[36] De Laet C, Thiry N, Holdt Henningsen K, Stordeur S, Camberlin C (2015) Percutaneous vertebroplasty and balloon kyphoplasty—synthesis. Health Technology Assessment (HTA) Brussels: Belgian Health Care Knowledge Centre (KCE). KCE Reports 255Cs. https://kce.fgov.be/sites/default/files/page_documents/KCE_255C_Percutaneaous_vertebroplasty_Synthesis.pdf

[37] National Institute for Health and Care Excellence (2013) Percutaneous vertebroplasty and percutaneous balloon kyphoplasty for treating osteoporotic vertebral compression fractures. nice.org.uk/guidance/ta279. Accessed May 26, 2017

[38] Swedish Council on Health Technology Assessment (2011) Percutaneous vertebroplasty and balloon kyphoplasty in treating painful osteoporotic vertebral compression fractures. https://www.ncbi.nlm.nih.gov/pubmedhealth/PMH0078702/pdf/PubMedHealth_PMH0078702.pdf. Accessed May 26, 2017 26153594

